# Prevalence of intimate partner violence in Malaysia and its associated factors: a systematic review

**DOI:** 10.1186/s12889-020-09587-4

**Published:** 2020-10-15

**Authors:** Hayati Kadir Shahar, Faridah Jafri, Nor Afiah Mohd Zulkefli, Norliza Ahmad

**Affiliations:** 1 Malaysian Research Institute of Ageing (MyAgeing), Universiti Putra Malaysia, 43400 Serdang, Selangor Darul Ehsan Malaysia; 2 Department of Community Health, Faculty of Medicine and Health Sciences, Universiti Putra Malaysia, 43400 UPM Serdang, Selangor Darul Ehsan Malaysia

**Keywords:** Intimate partner violence, IPV, Prevalence, Associated factors, Malaysia

## Abstract

**Background:**

Intimate partner violence (IPV) is any behaviour within an intimate relationship that causes physical, psychological or sexual harm to those in the relationship. IPV is an important public health problem with substantial consequences on physical, mental, sexual, and reproductive health. Data on the systematic review of IPV are vital as basis for policy and program recommendations. The purpose of this systematic review was to ascertain the prevalence of IPV and its associated factors in Malaysia.

**Materials and methods:**

A systematic review was conducted on published research studies from four databases which included Scopus, Medline, Sage and Google Scholar using keywords of intimate partner violence OR IPV AND associated factors OR risk factors OR protective factors AND Malaysia. Articles included were either cross-sectional, cohort or case-control studies which were published between the year 2005 till present. Excluded articles were the non-Malaysian origin, irrelevant topics being studied and articles not written in English.

**Results and discussion:**

Out of 1983 records identified and screened, five were included for the analysis and interpretation of the data. All of the included studies were of cross-sectional design in which one of the studies was secondary data. IPV prevalence in Malaysia has a wide range between 4.94 and 35.9%. Two studies reported emotional or psychological abuse as the most common form of IPV (13% out of 22%) and (29.8%; CI = [0.27, 0.32]). Significant factors associated with IPV were lower education background, lower socio-economic status, history/ current substance abuse, exposure to prior abuse or violence, violence-condoning attitude; husbands or partners controlling behaviour, substance abuse and involvement in fights and lack of social support.

**Conclusion:**

Specific IPV intervention should focus on lower socio-economic groups, high-risk institutionalised groups, the involvement of partners or husband and addressing issues of substance abuse.

## Background

World Health Organization (WHO) defines violence as the intentional use of physical force or power, threatened or actual; either against oneself, another person, a group or a community which results in/ has a high likelihood to result in injury, death, psychological harm, maldevelopment or deprivation [1]. Intimate partner violence (IPV) which refers to violence between couples, is defined in detail as any behaviour within an intimate relationship that causes physical, psychological or sexual harm to those in the relationship. The behaviour mentioned above includes acts of physical and sexual violence, emotional-psychological abuse and controlling behaviours. An intimate partner is a person with whom an individual has a close relationship which may be characterised by any of the followings; emotional connectedness, regular contact, ongoing physical contact and sexual behaviour, identity as a couple, with familiarity and knowledge about each other’s lives. Intimate partner relationships include current or former, spouses (either married or non-married partners), boyfriends/ girlfriends and ongoing sexual partners. Individuals in intimate partner relationships may or may not be cohabiting and can be of the opposite or same-sex [2]. It is worth to distinguish the difference between IPV and domestic violence in which the latter encompasses broader aspects of abuse which occur in a domestic relationship, such as child abuse, elderly abuse or abuse by any members of a household. The term “intimate partner” means violence can be perpetrated by both men or women, regardless of marital status, age or sexual orientation. However, the overwhelming global burden of IPV is borne by women as a result of unequal power relationships between men and women [3].

The worldwide prevalence of IPV among all ever-partnered women was 30.0% (95% CI 27.8 to 32.2%) with regions that reported the greatest number of cases were WHO African, Eastern Mediterranean and South-East Asia Regions. Lower prevalence of IPV was seen in the high-income regions such as European and Western Pacific regions which reported IPV prevalence rate between 23 and 25%. Prevalence of IPV among South-East Asian countries varied between 13.7% (95% CI 11.6–15.7) in Cambodia, 14.8% (95% CI 13.9–15.7) in The Philipines and 34.3% (95% CI 31.2–37.5) in Timor Leste [4]. In Malaysia, among the most recent evidence on the prevalence of IPV among all ever-partnered women were conducted by Shuib et al. [5] among households nationwide. The survey demonstrated the prevalence of IPV of approximately 8% among all ever-partnered women in Malaysia. However, the result could have been underestimated as the nature of IPV as being a sensitive topic and is usually under-reported. A 2010 statistic by Royal Malaysian Police Force revealed intimate partner violence constituted to more than 50% of acts of violence against women in Malaysia followed by rape, incest and abuse of domestic workers. It was also reported an escalating trend in the number of IPV reported cases from 3468 cases in the year 2000 to 5513 cases in the year 2017 [6].

A cultural study on family violence in Malaysia and Australia by Professor Dale Bagshaw from Hawke Research Institute’s Centre for Peace (2008) revealed that IPV occurs at all levels in the society and remained invisible due to the sensitive nature and fear of victims to report the acts of abuse [7]. IPV is still seen from a narrow perspective which mainly involves physical violence, which causes other forms of abuse being overlooked and neglected. It inevitably leads the victims to live in constant fear without acknowledging the acts of abuse that are being experienced. Being a Muslim majority country, which highly emphasises on the legality of relationships causes capturing of violence data only among intimate partners who either spouses or ex-spouses of legal relationships. It, however, underestimates the prevalence of IPV which occur outside the marriage institution.

Involve in IPV either as the perpetrators or victims, is negatively associated with physical as well as mental health. For women, IPV results in physical, sexual, mental harm or suffering, which includes threats, coercion and arbitrary deprivation on their freedom in public and private life. IPV is also linked to detrimental effects on the sexual reproductive health of women which include sexually transmitted infections such as HIV, miscarriages, unsafe deliveries, risky sexual behaviours [8]. Identifying risk and protective factors towards IPV is paramount in combating the problem at an earlier stage. It will assist in closing the information gap regarding the current situation of IPV as well as contributing to the prevention and control strategies. Many researchers have researched IPV and its associated and protective factors [9–11]. However, due to methodological differences and context-specific factors; the severity of IPV, prevalence and its associated factors may vary according to cultural-specific contexts. Kerley et al. (2010) utilised secondary data of 816 married women in urban Thailand; Oyunbileg et al. (2009) conducted household survey of 5500 mixed urban and rural Mongolian women whereas Coutinho et al. (2015) conducted a cross-sectional survey using convenient sampling from pregnant women at public hospitals in Portugal. Majority of the studies mentioned above emphasised on the individual, family and community characteristics in further understanding associated and protective factors of IPV, especially in a cultural-specific context. This review is aimed to determine the current prevalence of IPV and its associated factors in Malaysia.

## Main text

### Methodology

**Fig. 1:**
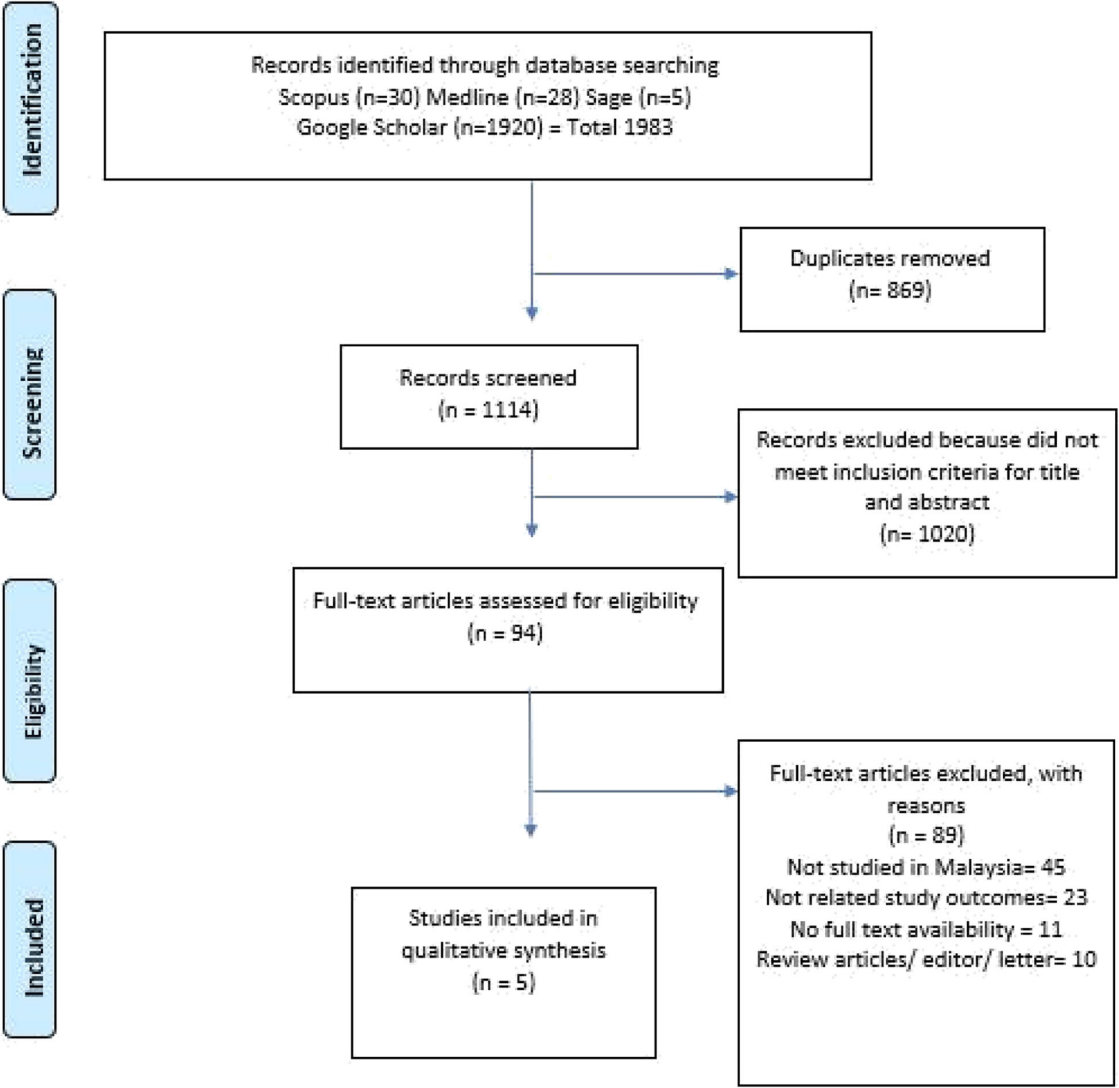
Flow diagram of the selection process used to identify studies for inclusion in this review based on PRISMA Statement

The review is adhered to the Preferred Reporting Items for Systematic Reviews and Meta-analyses (PRISMA) statement (Fig. 1) [12]. Articles included were observational studies either cross-sectional studies, cohort studies or case-control with publication period between the year 2005 till present. The main subject discussed in the articles was intimate partner violence which referred to violence within an intimate relationship perpetrated either by intimate partner/ spouse or ex-partner/ spouse. Articles which were excluded were those of non-Malaysian origin, with irrelevant topics being studied and articles which were not written in English. Official reports from government agencies were not selected as the review only included articles with both information on the prevalence and associated factors of IPV.

We searched four electronic databases from inception to June 2019, which included Scopus, Medline, Sage and Google Scholar to retrieve studies of potential interest. The literature search was undertaken using a combination of keywords of intimate partner violence OR IPV AND associated factors OR risk factors OR protective factors AND Malaysia in the abstract, title or keywords fields. All duplicates were removed electronically and then manually. The titles of the study were screened, and the abstract was analysed to determine its relevance.

### Study selection

Two researchers independently screened titles and abstracts based on inclusion and exclusion criteria. If the inclusion or exclusion criteria could not be decided based on the title and abstract, full-text articles were retrieved, and the decision was made accordingly. Criteria of studies that were sought for were study design, study location, sample size, objective of the studies and major findings such as the prevalence of IPV against women by their husband/intimate partner. The associated factors for IPV were extracted using 95% CI and odds ratio/adjusted odds ratio. Articles which used community-based studies, cross-sectional studies and secondary data which reported the prevalence and associated factors were included.

### Data extraction

After the screening of articles, duplicated publications were determined and excluded by comparing authors’ names, study names and sample size. Study design, sample size and the results of each study were noted. Results selected to be included in the review had to possess specific measure estimates either calculated crude odds ratio, adjusted odds ratio, relative risk ratio, standardised beta coefficient with 95% confidence interval that does not include one or a *p*-value of less than 0.05 to have a significant factor included.

Upon completion of screening and selection of the retrieved studies, methodological assessment of each study was conducted and followed by extraction of data. The data from the selected studies were extracted according to (1) study overview or characteristics of the study; (2) prevalence of IPV and the (3) associated factors or risk factors or protective factors.

### Risk of bias assessment

The Crowe Critical Appraisal Tool (CCAT) was used to assess the methodological quality of each observational study. The CCAT examined the study based on eight criteria which included preliminaries, introduction, design, sampling, data collection, ethical matters, result, discussion and conclusion. The total score was then converted into percentage whereby the following categories were assigned to allow for comparison; poor quality (≤50%), acceptable quality (51–74%), high quality (≥75%) [13].

## Results

### Study selection

A total of 1983 records were identified through four databases searching. A total of 1114 records remained after duplicates were removed. Of those remaining, 1020 records were deemed ineligible based on their titles and abstracts. Of the 94 papers that were qualified for a full-text review, 89 full-text articles were excluded because they did not meet the eligibility criteria for the review (see Fig. 1).

### General characteristics of the included studies

Five peer-reviewed papers were used for the analysis and interpretation of the data. All the selected studies included the prevalence of IPV and its associated factors as their main findings. General characteristics of the included studies and their study findings were summarised in Table 1. All of the five included studies were of cross-sectional design in which one of the studies was generated from secondary data [17]. Only two studies adopted probability sampling [14, 16] while the other three studies used either universal or convenient sampling method. Out of five included studies, four of them were conducted at government health settings (both primary health clinics and hospitals) [14–16, 18] while the other one study was generated from secondary data obtained from Malaysia Women’s Aid Organization [17]. In terms of the study population, two studies were conducted among general women population [14, 18] while another two studies were carried out among pregnant and post-partum women [15, 16].

**Table 1:** General characteristics and summary of the results of the included studies

Author/ year	Study design	Study location	Population sampling/ Sample size	Objective	Main findings
Othman, S., et al. (2019) [14]	Cross-sectional study	Six public primary care clinics in Kuala Lumpur	882 women aged 16 years and above recruited via systematic sampling	To assess the prevalence of IPV among women attending urban primary care clinics and to examine the associated risk factors	1. The prevalence of IPV in the past 12 months was estimated to be 22.0% (*n* = 194). Approximately 13% of them experienced psychological violence only, 0.7% reported assault only, and 8.6% experienced both. 2. Identified risk factors of IPV were women of Chinese ethnicity (AOR = 2.02, 95% CI = [1.30, 3.12]), household income of RM3,000 (AOR = 2.04; 95% CI = [1.32, 3.16]), women who have witnessed parental IPV (AOR = 3.52; 95% CI = [2.29,5.41]) and those who experienced poor psychological well-being (AOR = 2.16; 95% CI = [1.38, 3.38]).
Haron, K., et al. (2018) [15]	Cross-sectional study	A hospital in the northern state of Peninsular Malaysia	1200 post-natal women aged 18 years and above recruited via universal sampling	To determine the prevalence of men’s violence against pregnant women and its association with women’s attitude	1. Prevalence of men’s violence against pregnant women was 35.9% (CI = [0.33, 0.39]), with emotional violence the commonest (29.8%; CI = [0.27, 0.32]), followed by physical violence (12.9%; CI = [0.11, 0.15]) and sexual violence (9.8%; CI = [0.08, 0.12]). 2. Women who were drug users appeared as a risk factor for both EV and PV with AOR of 6.96 and 43.66, respectively 3. Being exposed to violence during childhood was another risk factor identified for EV (AOR 1.52 95% CI = [1.05, 2.09]) 4. Multipara women (having two or more children) were more likely to experience SV during pregnancy (AOR 1.5 95% CI = [1.03, 2.17]) 5. Women’s attitude which condones patriarchal supremacy, justification of husbands to hit wives and justification of wives to refuse sex on certain conditions were more likely to experience any types of violence during pregnancy (AOR 1.47 95% CI = 1.1, 1.98); (AOR 1.93 95% CI = 1.51, 2.59); (AOR 1.89 95% CI = 1.31, 2.72) respectively.
Chan, Y., Y., et al. (2019) [16]	Cross-sectional study	106 government primary health care clinics in 16 states within Malaysia	6669 women between 6 to 16 weeks post-partum, aged 18 years and above recruited via random cluster sampling	To determine the prevalence and factors associated with lifetime and past-year IPV among post-partum women	1. The overall prevalence of lifetime and past-year IPV among post-partum women in this study were 4.94% (95% CI [3.81,6.39]) and 2.42% (95% CI [1.74,3.35]) respectively. 2. Husband’s/partner’s behavioral factors significantly associated with a higher likelihood of lifetime IPV are frequent alcohol drinking (AOR = 9.11, 95% CI [2.44, 34.04]), drug use (AOR = 5.70, 95% CI [1.25, 26.07]), involvement in physical fights (AOR = 23.48, 95% CI [8.65, 63.76]) and controlling behaviors (AOR = 2.77, 95% CI [1.44, 5.33]). 3. Chinese women were significantly less likely to report the experience of past-year IPV compared to Malay women (AOR = 0.18, 95% CI [0.04,0.82]). Post-partum women who were currently not married/ no current partner were significantly more likely to have experienced IPV in the past year compared to those who were currently married/ has a partner (AOR = 11.27, 95% CI [2.26,56.17]). 4. Husband’ s/partner’s behavioural factors were all significantly associated with a higher likelihood of women experiencing past-year IPV; frequent alcohol drinking (AOR = 10.37, 95% CI [2.96, 36.33]), drug use (AOR = 9.55, 95% CI [3.48, 26.18]), involvement in physical fights (AOR = 10.81, 95% CI [3.60, 32.49]) and controlling behaviours (AOR = 5.90, 95%CI [2.70, 12.86]).
Awang, H., et al. (2011) [17]	A cross-sectional study based on secondary data	Secondary data obtained from Malaysia Women’s Aid Organization (WAO)	164 case files of women who sought shelter services from 2002 to 2005	To investigate the extent & determinants of domestic violence within a multi-ethnic society in Malaysia	1. Frequency of abuse: 26% of the survivors being abused nearly daily, 37% abused up to three times a week, and 26% are victims of an unpredictable frequency of abuse 2. Significant protective factors for nearly daily of abuse: Age of perpetrator between 30 and 39 (OR 0.375, *p* = 0.064); Nil income of survivor (OR 0.311, *p* = 0.018). 3. Significant protective factors for 1–3 times of abuse a week: Number of children:1–3 child (OR 0.441, *p* = 0.088) 4. Significant risk factor for once or twice of abuse a month: Age of perpetrator: ≤29 years Age (OR 15.337, *p* = 0.019)
Yut-Lin, W., et al. (2008) [18]	Cross-sectional study	Eight primary health centres in Selangor	710 female patients above 16 years of age recruited via convenient sampling	To identify domestic violence and its prevalence among adult women patients attending the primary care clinics To determine the relationship between social correlates such as income, education, ethnicity, location (urban/rural) and residence of adult patients, and domestic violence screening	1. Prevalence of domestic violence = 5.6% measured through the WAST screening score 2. Significant factors associated with domestic violence were Indian ethnicity (χ2 = 24.247, df = 3, *P* < 0.001); low income group were at greater risk of experiencing DV (χ2 = 8.812, df = 2, *P* = 0.012), lower education level of both victims and partners (χ2 = 14.398, df = 3, *P* = 0.002; χ2 = 22.788, df = 4, *P* < .0.001) respectively.

### Quality assessment

Quality assessment for each study was conducted using Crowe Critical Analysis Tool; an established and validated tool used in assessing the quality of observational studies [13]. Two studies were rated to be of high quality, while the other three studies were of acceptable quality (Table 2) [19].

**Table 2:** Quality assessment of studies using Crowe Critical Analysis Tool (CCAT)

Category		Othman, S., et al. (2019) [14]	Haron, K. et al. (2018) [15]	Chan, Y., Y., (2019) [16]	Awang, H., et al. (2011) [17]	Yut-Lin, W., et al. (2008) [18]
1.	Preliminaries (/5)	4	4	4	3	4
2.	Introduction (/5)	4	4	4	3	4
3.	Design (/5)	4	3	4	3	3
4.	Sampling (/5)	4	3	4	3	3
5.	Data collection (/5)	4	4	4	3	4
6.	Ethical matters (/5)	4	4	4	2	3
7.	Results (/5)	4	3	3	3	4
8.	Discussion (/5)	4	4	4	3	4
9.	Total score (/40)	32	29	31	23	29
10.	Percentage (%)	80	72.5	77.5	57.5	72.5

## Main findings

Findings from five included studies demonstrated a wide range of IPV prevalence between 4.94 to 35.9%. Two studies reported emotional or psychological abuse as the most common form of IPV (13% out of 22%) [14, 15] (29.8%; CI = [0.27, 0.32]). One study reported both life-time prevalence of IPV and past-year prevalence of IPV which was reported as 4.94% (95% CI [3.81,6.39]) and 2.42% (95% CI [1.74,3.35]) respectively [16]. A detailed description on the frequency of IPV among victims was described by Awang et al. [17] whereby 26% of the survivors were being abused nearly daily, 37% abused up to three times a week, and 26% were victims of an unpredictable frequency of abuse.

Review findings showed that factors associated with IPV were multi-faceted and could be represented further using Ecological Model which proposes that violence is a result of factors operating at four levels: individual, relationship, community and societal [20].

At the individual level, significant factors found were ethnicity, level of education, exposure to other forms of prior abuse and women’s attitude towards violence [14, 18]. Women of Chinese ethnicity are two times more likely to experience IPV compared with women of Malay ethnicity (AOR = 2.02, 95% CI = [1.30, 3.12]) [14] while a study by Yut-Lin W et al. [18] reported Indians were more likely to experience domestic violence than the others (χ2 = 24.247, df = 3, *P* < 0.001). Lower education level of both perpetrators and victims put them at greater risk of experiencing DV (χ2 = 14.398, df = 3, *P* = 0.002; χ2 = 22.788, df = 4, *P* < .0.001 respectively). Women who have witnessed parental IPV (AOR = 3.52; 95% CI = [2.29,5.41]) and being exposed to violence during childhood was another risk factor identified for emotional violence (AOR 1.52 95% CI = [1.05, 2.09]). Women’s attitude which condones patriarchal supremacy, justification of husbands to hit wives and justification of wives to refuse sex were more likely to experience any types of violence during pregnancy (AOR 1.47 95% CI = 1.1, 1.98); (AOR 1.93 95% CI = 1.51, 2.59); (AOR 1.89 95% CI = 1.31, 2.72) respectively.

At relationship level, significant factors associated with IPV were household income and husband’s or partner’s behavior. Household income of RM3,000 or less was associated with IPV (AOR = 2.04; 95% CI = [1.32, 3.16]) [14] and those from low income group were at greater risk of experiencing intimate partner violence (χ2 = 8.812, df = 2, *P* = 0.012) [14, 21]. Husband’s/partner’s behavioral factors significantly associated with a higher likelihood of lifetime IPV are frequent alcohol drinking (AOR = 9.11, 95% CI [2.44, 34.04]), drug use (AOR = 5.70, 95% CI [1.25, 26.07]), involvement in physical fights (AOR = 23.48, 95% CI [8.65, 63.76]) and controlling behaviors (AOR = 2.77, 95% CI [1.44, 5.33]).

At the community or societal level, the only significant factor associated with IPV from this review was social support. Women who have less social support (AOR = 0.985; 95% CI = [0.973, 0.997] have a higher likelihood of experiencing IPV [14].

## Discussion

Determination of the prevalence of intimate partner violence and their related factors in Malaysia was explored in this research. A wide spectrum of prevalence was shown in this study, with IPV prevalence ranging from 4.94% to 35.9%. The broad differences in IPV prevalence may have derived from a variety of potential causes. These include the use of a long-term system from 2005 to the present, the use of different sample populations that may have contributed to several problems (two studies were conducted within the general female population, the other two studies were conducted between antenatal and post-partum women) and the use of different survey instruments. For example, among the different tools used in this review were Women Abuse Screening Tool (WAST), Women’s Experience with Battering Scale (WEB-scale), Women’s Health and Life Experiences questionnaire and WHO Multi-Country Study on Women’s Health and Life Events Questionnaire. WAST consisted of eight items with questions about socio-economic background, family well-being, and women’s health. All the items were related to, or predictive of, woman abuse with most respondents reported that WAST screening questions were clear and easily comprehensible. WEB-scale is a 10-item questionnaire which assesses women’s experience with battering scale. It operationalizes the experiences of battered women rather than the nature of the physical or psychological abuse they encounter. It has good internal consistency, with a reported Cronbach’s alpha coefficient of 0.93 [14]. WHO Multi-Country Study on Women’s Health and Life Events Questionnaire was prepared in bilingual forms with validation done locally. It is comprehensive as it assesses all types of violence, which include psychological, physical, and sexual violence.

The locally validated and comprehensive tool is recommended for use among the Malaysian population as it represents the experience of IPV among women in Malaysia. Also, the use of non-standardised data collection method may have contributed to the variation in the findings whereby studies which utilised self-administered questionnaire reported a higher prevalence of IPV as compared to those with face-to-face interviews. It explained the intricate nature of the topic, which respondents were more willing to disclose information at their discretion. It is duly important to note that both past-year IPV and lifetime IPV prevalence were two distinctive figures which were greatly affected by recall bias. The relatively low prevalence of IPV in the Malaysian community may have resulted from the under-reporting of such cases due to its sensitive nature. A 2008 nationwide study among 1000 Malay women on perceptions on women’s roles and progress demonstrated women were perceived to have primary roles towards the family by supporting the husband or nurturing the children. Also, women were expected to maintain their femininity, be subservient to the husband and willing to make sacrifices when necessary [22]. These patriarchal values which are still firmly rooted in the Muslim-majority society may influence the reactions of women when faced with marital dispute or disharmony. The lifetime prevalence of IPV, as reported in this review, was higher as compared to other Asian countries such as Singapore at 9.2% and Japan at 15.4%. The review finding was, however, lower as compared to IPV prevalence in Ethiopia (which ranged from 50 to 78%) [7], Thailand at 41.1%, and Bangladesh at 53.4% [23]. The results were in line with the 2013 report by WHO which revealed a higher prevalence of IPV among lower to middle-income countries as compared to the more affluent counterparts [24]. The economic status of a country population may have exerted impacts on women empowerment, improved literacy rate and financial dependency of women and a more gender equitable norm among the society.

Two studies in this review highlighted psychological or emotional abuse as the most prevalent form of IPV inflicted on women followed by physical and sexual abuse. The low prevalence of physical and sexual violence may, however, be due to under-reporting from cultural factors or traditional norms which accept wife-beating at certain conditions. However, being a majority Malay-Muslim nation, traditional male-dominated culture still exists despite the country’s modernisation, which may have prevented women from speaking out and exposed their marital problems. The prevalence of IPV in Malaysia is, however, lower than other Muslim majority countries such as Palestine (42.5%), Bangladesh (31.9%) and Saudi Arabia (43%) [25–27]. This finding could be explained by greater male dominance in the latter countries which regard marital violence as legitimate and acceptable in their culture. Male domination is, however, not only confined to the Malay-Muslim community in Malaysia, but it also occurs in other major ethnic groups. The status of women in modern Malaysian Chinese and Malaysian Indian communities may be more closely tied to the traditional cultures of East and South Asia that were allegedly more patriarchal than those of South-east Asia. The finding was in agreement with a systematic review on the worldwide prevalence of domestic violence against women which reported emotional violence as the most common form of IPV across all continents worldwide (mean prevalence ranged from 37 to 78%) [28].

Factors associated with IPV originated from multiple levels in the society as demonstrated in this review which included individual, relationship, community and societal level. This finding is in agreement with other multi-country studies [29, 30] which emphasised the interplay of factors that exist at different societal levels. At an individual level, those who were at greater risks of experiencing IPV were more likely to be from a lower education background, from lower socio-economic status, had history/ current substance abuse, had exposure to other forms of prior abuse or violence and possessed violence-condoning attitude. The correlation between lower socio-economic status and higher prevalence of either perpetration or victimisation of IPV may be explained by a lack of access to resources and greater acceptance towards violence [28]. The previous history of abuse reinforces the normative nature of violence thus increases the likelihood of male perpetration and women’s acceptance of violence [31].

At the relationship level, husband’s or partner’s behaviour such as history or current substance abuse, involvement in fights, controlling behaviour was associated with greater risk of IPV while at the societal level, lesser social support places individuals at greater risks of experiencing IPV. The misuse of the substance may have precipitated act of violence via disruption of the thinking process, a manifestation of power and control and hostile personality [32]. The findings above recognised that there was no single factor that could explain the complexity of why some women were at higher risk of IPV than the others, and IPV reduction strategies should then call for cross-sectorial efforts from stakeholders at each level in the society.

Three studies included in this review were based on nationwide data which may have improved the generalizability of the review findings. However, few limitations were identified among which our search focused on literature published in the English language only, which may have missed those published in the Malay language. All of the included studies were of cross-sectional design which may be limited in determining causality.

Four out five studies were conducted in health facilities which may have introduced selection bias, as there might be more cases that could have been detected in the general community. Social desirability bias was also an issue as the subject discussed in the review was a sensitive topic, and respondents tended to give answers that conformed to social and cultural expectations.

## Conclusion

IPV prevalence in Malaysia has a wide range between 4.94 and 35.9%. Emotional or psychological violence was recognised as the most common form of IPV, followed by physical and sexual violence. Significant factors associated with IPV were lower education background, lower socio-economic status, had history/ current substance abuse, exposure to other forms of prior abuse or violence, violence-condoning attitude; husbands or partners controlling behaviour, substance abuse and involvement in fights and lack of social support. This review provides important information regarding common factors of IPV that need to be addressed. Specific IPV intervention should focus on lower socio-economic groups and high-risk groups such as institutionalised children or adolescents to correct their attitude on violence and improve help-seeking behaviour. Involvement of partners or husband and addressing issues of substance abuse in IPV intervention strategies are vital in which the curriculum may be integrated into pre-marital courses.

## Data Availability

The datasets used and/or analysed during the current study are available from the corresponding author on reasonable request.

## Abbreviations

IPV: Intimate partner violence
WHO: World Health Organization
PRISMA: Preferred Reporting Items for Systematic Reviews and Meta-analyses
CCAT: The Crowe Critical Appraisal Tool
AOR: Adjusted Odds Ratio
WAST: Women Abuse Screening Tool
WEB-scale: Women’s Experience with Battering Scale
